# Characterization of KPC-Producing *Serratia marcescens* in an Intensive Care Unit of a Brazilian Tertiary Hospital

**DOI:** 10.3389/fmicb.2020.00956

**Published:** 2020-05-20

**Authors:** Roumayne L. Ferreira, Graziela S. Rezende, Marcelo Silva Folhas Damas, Mariana Oliveira-Silva, André Pitondo-Silva, Márcia C. A. Brito, Eduardo Leonardecz, Fabiana R. de Góes, Emeline Boni Campanini, Iran Malavazi, Anderson F. da Cunha, Maria-Cristina da Silva Pranchevicius

**Affiliations:** ^1^Departamento de Genética e Evolução, Universidade Federal de São Carlos, São Carlos, Brazil; ^2^Programas de Pós-graduação em Odontologia e Tecnologia Ambiental, Universidade de Ribeirão Preto, Ribeirão Preto, Brazil; ^3^Laboratório Central de Saúde Pública do Tocantins, Palmas, Brazil; ^4^Instituto de Ciências Matemáticas e de Computação, Universidade de São Paulo, São Carlos, Brazil

**Keywords:** *Serratia marcescens*, intensive care units, KPC, virulence and resistance genes, ERIC-PCR

## Abstract

*Serratia marcescens* has emerged as an important opportunistic pathogen responsible for nosocomial and severe infections. Here, we determined phenotypic and molecular characteristics of 54 *S. marcescens* isolates obtained from patient samples from intensive-care-unit (ICU) and neonatal intensive-care-unit (NIUC) of a Brazilian tertiary hospital. All isolates were resistant to beta-lactam group antibiotics, and 92.6% (50/54) were not susceptible to tigecycline. Furthermore, 96.3% showed intrinsic resistance to polymyxin E (colistin), a last-resort antibiotic for the treatment of infections caused by MDR (multidrug-resistant) Gram-negative bacteria. In contrast, high susceptibility to other antibiotics such as fluoroquinolones (81.5%), and to aminoglycosides (as gentamicin 81.5%, and amikacin 85.2%) was found. Of all isolates, 24.1% were classified as MDR. The presence of resistance and virulence genes were examined by PCR and sequencing. All isolates carried KPC-carbapenemase (*bla*_*KPC*_) and extended spectrum beta-lactamase *bla*_*TEM*_ genes, 14.8% carried *bla*_*OXA–*__1_, and 16.7% carried *bla*_*CTX–M–*__1__*group*_ genes, suggesting that bacterial resistance to β-lactam antibiotics found may be associated with these genes. The genes *Sde*B/*Has*F and *Sde*Y/*Has*F that are associated with efflux pump mediated drug extrusion to fluoroquinolones and tigecycline, respectively, were found in 88.9%. The aac(6′)-Ib-cr variant gene that can simultaneously induce resistance to aminoglycoside and fluoroquinolone was present in 24.1% of the isolates. Notably, the virulence genes to (i) pore-forming toxin (*Shl*A); (ii) phospholipase with hemolytic and cytolytic activities (*Phl*A); (iii) flagellar transcriptional regulator (*Flh*D); and (iv) positive regulator of prodigiosin and serratamolide production (*Pig*P) were present in 98.2%. The genetic relationship among the isolates determined by ERIC-PCR demonstrated that the vast majority of isolates were grouped in a single cluster with 86.4% genetic similarity. In addition, many isolates showed 100% genetic similarity to each other, suggesting that the *S. marcescens* that circulate in this ICU are closely related. Our results suggest that the antimicrobial resistance to many drugs currently used to treat ICU and NIUC patients, associated with the high frequency of resistance and virulence genes is a worrisome phenomenon. Our findings emphasize the importance of active surveillance plans for infection control and to prevent dissemination of these strains.

## Introduction

*Serratia marcescens* is a Gram-negative bacillus that naturally resides in the soil and water and produces a red pigment at room temperature. Although previously considered non-pathogenic, this species has emerged as a prominent opportunistic pathogen found in nosocomial outbreaks in neonatal intensive care Units (NICUs), intensive care units (ICUs) and other hospital units over the last few decades (Enciso-Moreno et al., 2004; Moradigaravand et al., 2016; Ghaith et al., 2018).

The true occurrence of *S. marcescens* is still underestimated (Zingg et al., 2017). In NICUs, studies have showen that infected newborns are a potential source of *S. marcescens* (Cristina et al., 2019), although there is a constant increase of *S. marcescens* bacteremia across all age groups (Vetter et al., 2016; Phan et al., 2018). *S. marcescens* increasingly adapts to hospital environments (Yoon et al., 2005; Gastmeier, 2014). It accounts for 15% of all isolates from nosocomial infections (Raymond and Aujard, 2000). Although it is difficult to identify the source of *S. marcescens* during outbreaks, it is the third most frequent pathogen identified (Gastmeier et al., 2007), and more than one clone can be usually identified (David et al., 2006; Montagnani et al., 2015; Dawczynski et al., 2016).

*Serratia marcescens* associated with hospital outbreaks or epidemic events are commonly resistant to several antibiotics (Moradigaravand et al., 2016; Cristina et al., 2019). In fact, one important feature of *S. marcescens* is its resistance to narrow-spectrum penicillins and cephalosporins; nitrofurantoin; tetracycline; macrolides; cefuroxime; cephamycins; fluoroquinolone, and colistin (Stock et al., 2003; Liou et al., 2014; Moradigaravand et al., 2016; Sandner-Miranda et al., 2018). The resistance to some of these molecules may be intrinsic to this specie and is explained by either the presence of resistance genes on the chromosome or by the acquisition of such genes via horizontal transfer. It is noteworthy that the latter mechanism is considered the most important event that leads to multiple antibiotic resistance (von Wintersdorff et al., 2016; Sandner-Miranda et al., 2018).

Extended-spectrum β-lactamases (ESBLs) are a group of bacterial enzymes that can be rapidly transferred via plasmid exchange (Rawat and Nair, 2010) causing resistance to a broad range of β-lactams (Naas et al., 2008). Carbapenemases are the most versatile family of β-lactamases able to hydrolyze carbapenems and many other β-lactams (Jeon et al., 2015) including penicillins, cephalosporins, and monobactams (Anderson et al., 2007; Marschall et al., 2009). In general, bacteria carrying the *bla*_*KPC*_ and/or ESBLs genes usually harbor other resistance genes associated with several classes of antimicrobials (Tzouvelekis et al., 2012; Cao et al., 2014; Ribeiro et al., 2016). Since *S. marcescens* has been acquiring a range of ESBLs and commonly exhibit co-resistance to many other classes of antibiotics, the infections caused by these multidrug-resistant (MDR) isolates impair therapy and limit treatment options (Yu et al., 1998; Mostatabi et al., 2013; Herra and Falkiner, 2018).

In this study, we investigated the phenotypic characteristics regarding antimicrobial resistance and the genotypic traits of *S. marcescens* isolated from a tertiary care hospital’s ICUs including the search for resistance and virulence genes as well as the genetic relationship among the isolates. Our report describes MDR profile and KPC-producing *S. marcescens* isolates and highlight the importance of monitoring *S. marcescens* infection and the need of constant surveillance to support continuous and effective measures to prevent the spread of these strains.

## Materials and Methods

### Study Design and Bacterial Isolates

From February 2014 to June 2015, a total of 54 *S. marcescens* were isolated of clinical specimens collected from 45 patients admitted to intensive-care-unit (ICU) and neonatal intensive care unit (NICU) of a tertiary care government hospital in Palmas, Tocantins, Brazil. Since 2013, there has been an increase in detection of *S. marcescens* isolates from hospital inpatients, and in 2015, the hospital reported an apparent *S. marcescens* outbreak that occurred from July to August 2015. Appropriate intervention measures were established, such as reviewing the infection control policies, hand antisepsis practices and determination of trends of isolation of *S. marcescens* over time. To trace the source of the infection, bacteria were isolated from various samples obtained from clinical indications of infections during the patients’ ICU stay.

As part of the control measures, surveillance cultures were obtained from tracheal aspirate and rectal swabs from all ICU patients, on admission (within the first 24 h) and during the stay (once a week). Blood, wound, catheter tip, drain, sputum, urine, rectal swab, and tracheal aspirate samples were primarily sent to the hospital’s laboratory, processed and cultured by standard microbiological techniques. The blood samples were inoculated first in blood culture bottles (Hemoprov-NewProv, Brazil). All clinical samples, including blood culture bottles giving positive signals were cultured onto MacConkey agar (Probac, Brazil), blood agar (Probac, Brazil), and chocolate blood agar (Probac, Brazil). Plates were incubated at 37°C for up to 48 h. *S. marcescens* were identified by Gram staining, cultural characteristics in MacConkey agar (Probac, Brazil), blood agar (Probac, Brazil), and biochemical tests (Bactray I, II, III; Laborclin, Brazil). The antimicrobial susceptibility profile was determined by Kirby-Bauer disk diffusion method. All *S. marcescens* isolates and the microbiological reports prepared at the hospital were sent to the Central Laboratory of Public Health of Tocantins (LACEN-TO) for further phenotypic validations.

### Bacterial Identification and Antimicrobial Susceptibility Test

Once samples were received at LACEN, bacterial identification and antimicrobial susceptibility tests were performed by the Vitek 2 system (Biomerieux, France), according to Clinical and Laboratory Standards Institute guidelines (CLSI, 2019). All 54 *S. marcescens* isolates were screened for susceptibility against 16 antimicrobial agents: ampicillin (AMP), ampicillin/sulbactam (SAM), piperacillin/tazobactam (TZP), cefuroxime (CXM), cefoxitin (FOX), ceftazidime (CAZ), ceftriaxone (CRO), cefepime (FEP), ertapenem (ETP), imipenem (IPM), meropenem (MEM), amikacin (AMK), gentamicin (GEN), ciprofloxacin (CIP), tigecycline (TGC), and colistin (CST). Broth microdilution method was performed to determine tigecycline and colistin minimum inhibitory concentration (MICs) and results were interpreted based on the European Committee on Antimicrobial Susceptibility Testing (EUCAST, 2018) criteria, available at https://www.eucast.org/fileadmin/src/media/PDFs/EUCAST_files/Breakpoint_tables/v_8.1_Breakpoint_Tables.pdf. All isolates were tested for carbapenemase production by Modified Hodge test, synergy test and ethylenediaminetetraacetic acid (EDTA) test under the CLSI guidelines (CLSI, 2019) as described elsewhere (Miriagou et al., 2010; Nordmann et al., 2011; Okoche et al., 2015; Ferreira et al., 2019). Multidrug-resistance *S. marcescens* isolates were classified by non-susceptibility to at least one agent of three or more antimicrobial categories (Magiorakos et al., 2012). *S. marcescens* is intrinsically resistant to AMP, SAM, CXM, FOX, and CST; therefore, these antibiotics were not included in the MDR classification (Magiorakos et al., 2012).

### Genomic DNA Extraction

Isolates of *S. marcescens* were subcultured on Brain Heart Infusion (BHI) agar (Oxoid, United Kingdom) and incubated for 24 h at 37°C. All samples were submitted to genomic DNA extraction using the Wizard Genomic DNA Purification Kit (Promega, Madison, WI, United States), according to manufacture’s instructions.

### Detection of Antibiotic-Resistance

Polymerase chain reaction (PCR) was performed for detection of β-lactamase genes (*bla*_*TEM*_, *bla*_*SHV* variants_, *bla*_*OXA–*__1_, _4 and 30_, *bla*_*CTX–M–*__1 group_), carbapenemase genes (*bla*_*KPC*_, *bla*_*IMP*_, *bla*_*VIM*_, *bla*_*NDM*_, *bla*_*OXA–*__48_) (Ferreira et al., 2019), plasmid mediated quinolone resistance (PMQR) gene (*aac*(6’)-Ib-cr) (Wong et al., 2014; Mitra et al., 2019), resistance-nodulation-division (RND) efflux pumps (*Sde*B, *Sde*Y), and outer membrane gene (*Has*F, a *Tol*C homolog) involved in energy-dependent efflux of antimicrobial agents (Kumar and Worobec, 2005b). The genes were amplified using specific primers designed to follow the conditions described in the references from Table 1. All primers were synthesized by Exxtend (Brazil). Amplicons were analyzed by gel electrophoresis in 1.0% agarose and visualized under ultraviolet (UV) light.

**TABLE 1:** Sequences of primes used for detection of resistance markers.

**Gene**	**Sequence (5′-3′), F/R**	**TM (°C)**	**Amplicon size (bp)**	**References**
*bla*_*KPC*_	CGTCTAGTTCTGCTGTCTTG	61.3	797	Poirel et al., 2011
	CTTGTCATCCTTGTTAGGCG			
*bla*_*TEM*_	TGCGGTATTATCCCGTGTTG	63	296	Xiong et al., 2007
	TCGTCGTTTGGTATGGCTTC			
*bla*_*CTX–M–1 group*_,	ACAGCGATAACGTGGCGATG	64	216	Li and Li, 2005
	TCGCCCAATGCTTTACCCAG			
*bla*_*SHVvariants*_	AGCCGCTTGAGCAAATTAAAC	55.6	712	Dallenne et al., 2010
	ATCCCGCAGATAAATCACCAC			
*bla*_*OXA–1*_	GGCACCAGATTCAACTTTCAAG	63	563	Dallenne et al., 2010
	GACCCCAAGTTTCCTGTAAGTG			
*bla*_*OXA–48*_	GCGTGGTTAAGGATGAACAC	55	438	Poirel et al., 2011
	CATCAAGTTCAACCCAACCG			
*bla*_*IMP*_	CTACCGCAGCAGAGTCTTTGC	55	587	Martins et al., 2007
	ACAACCAGTTTTGCCTTACC			
*bla*_*VIM*_	AAAGTTATGCCGCACTCACC	55	865	Yan et al., 2001
	TGCAACTTCATGTTATGCCG			
*bla*_*NDM*_	GCAGCTTGTCGGCCATGCGGGC	60	782	Doyle et al., 2012
	GGTCGCGAAGCTGAGCACCGCAT			
*mcr*-1	CGGTCAGTCCGTTTGTTC	51.6	309	Liu et al., 2015
	CTTGGTCGGTCTGTAGGG			
*aac(6′)-Ib-cr*	ATGACTGAGCATGACCTTGC	55.4	519	Platell et al., 2011
	TTAGGCATCACTGCGTGTTC			
*Sde*B	AGATGGCCGATAAGCTGTTG	55.4	200	Hornsey et al., 2010
	CAGCGTCCAGCTTTCATACA			
*SdeY*	TCCATCAACGAAGTGGTGAA	55.5	200	Hornsey et al., 2010
	GTTTATCGAGAAGCCGAACG			
*Has*F	CATGTCGAAATGGCGCCAAC	57.5	785	Hornsey et al., 2010
	TTGTAGGCGTTGATGCTGCT			
*Pig*P	GAACATGTTGGCAATGAAAA	53.4	207	Srinivasan et al., 2017
	ATGTAACCCAGGAATTGCAC			
*Flh*D	TGTCGGGATGGGGAATATGG	57	307	Salini and Pandian, 2015
	CGATAGCTCTTGCAGTAAATGG			
*Shl*A	AGCGTGATCCTCAACGAAGT	55.4	217	Aggarwal et al., 2017
	TGCGATTATCCAGAGTGCTG			
*Phl*A	GGGGACAACAATCTCAGGA	55.4	207	Aggarwal et al., 2017
	ACGCCAACAACATACTGCTTG			

### Virulence Gene Detection

The presence of four virulence genes were assessed by PCR: genes *Pig*P, a positive regulator of prodigiosin and serratamolide production; *Flh*D, a flagellar transcriptional regulator; *Shl*A, a pore-forming toxin with hemolytic activity; *Phl*A, a phospholipase A with hemolytic activity. The primers sequences amplicon sizes and annealing temperatures are listed in Table 1. Amplicons were analyzed by gel electrophoresis in 1.0% agarose and visualized under ultraviolet (UV) light.

### Sequence Analysis of Antibiotic-Resistance Markers and Virulence Genes

One amplicon of each studied gene was randomly selected for confirmation of identity by DNA sequencing using an automated sequencer (ABI 3500xL Genetic Analyzer; Applied Biosystems, Foster City, CA, United States). After amplification, we extracted the PCR products from agarose gels using the Illustra GFX PCR DNA (GE Healthcare), which were purified using the Gel Band Purification Kit (GE Healthcare), both according to manufacturer’s instructions. Obtained sequences were edited with Bioedit v7.0.5 (Hall, 1999), compared with the nr database using the Blastn tool^1^ and submitted to the GenBank database. Genes and their respective accession numbers: *bla*_*CTX*_ – MK576103; *bla*_*KPC*_ – MK576104; *bla*_*OXA*_ – MK576105; *bla*_*TEM*_ – MK576106; *Sde*B – MN583232; *Sde*Y – MN583233; *Has*F – MN583234; *aac*(6′)-Ib-cr – MN583235; *Flh*D – MN583236; *Pig*P – MN583237; *Shl*A – MN583238; *Phl*A – MN583239). Access to genetic heritage was approved by the National System for the Management of Genetic Heritage (SisGen n° AFF27ED).

### Enterobacterial Repetitive Intergenic Consensus Polymerase Chain Reaction

Enterobacterial repetitive intergenic consensus PCR (ERIC-PCR) analysis was performed to evaluate the genetic similarity among the 54 *S. marcescens* isolates using the primers and conditions previously described by Versalovic et al. (1994). PCR reactions were performed using the enzyme TaKaRa Ex Taq DNA Polymerase (Takara Bio, Kusatsu, Japan). The BioNumerics program version 5.1 (AppliedMaths, Keistraat, Belgium) was used to construct the unweighted pair group mean method (UPGMA) similarity dendrogram with Dice’s similarity coefficient, following Ferreira et al. (2019).

### Statistical Analyses

In the analysis of contingency tables, we used Fisher’s exact test and/or Barnard’s exact test. Maximum likelihood did not present superior efficiency in relation to the previous methods (data not show). It was used logistic regression model with two predictor variables *x*_1_ and *x*_2_. Statistical software R was used in all data analysis.

### Ethical Considerations

In our study, we did not use/collect human genetic material and biological samples. Strains were part of the collection of the Central Laboratory of Public Health, (LACEN-TO), a health-care facility that is a reference in diagnosis in the state of Tocantins, Brazil. It was a retrospective study, and epidemiological data were obtained from a database or similar, which will be kept confidential in accordance with the with the terms of Resolution 466/12 of the National Health Council. These epidemiological data were also provided by LACEN-TO. Informed consent was not required according to resolution 466/12 concerning research involving humans of the National Health Council (Conselho Nacional de Saúde/Ministério da Saúde, Brasília, Brazil, 2012). The study was approved by the Committee of Ethics in Human Research of the Federal University of São Carlos (no. 1.088.936). Permission to conduct the study was also obtained from the Health Department of the State of Tocantins (Secretaria de Saúde do Estado do Tocantins – SESAU) and LACEN/TO.

## Results

### *Serratia marcescens* Isolates

**FIGURE 1:**
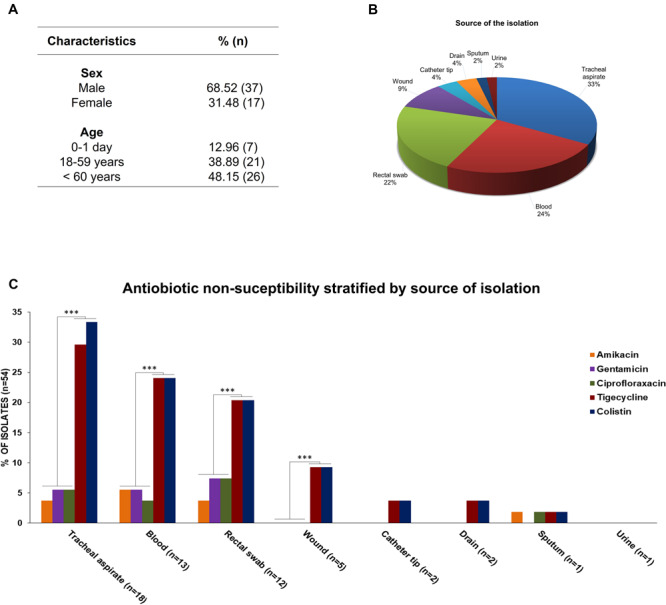
**(A)** Demographic characteristics of patients. **(B)** Percentage of *S. marcescens* per site of isolation. **(C)** Antibiotic non-susceptibility pattern of *S. marcescens* per site of isolation. *** statistical significance, *p*-value < 0.01.

A total of 54 *S. marcescens* strains were isolated from 39 ICU and 6 NICU patients’ samples at a tertiary hospital located in city of Palmas, Tocantins state. In six patients, 5 from ICU and 1 from NCIU, *S. marcescens* was isolated in more than one infection site. The prevalence of *S. marcescens* strains by age group was the following: 0–1 day (12.96%; *n* = 7), 18–59 years (38.89%, *n* = 21), 60 years or more (48.15%, *n* = 26). The median age of patients was 57.0 years (range, 0–93 years). *S. marcescens* strains were more frequently found in male (68.5%, *n* = 37) than in female (31.5%, *n* = 17) patients (Figure 1A). Forty-three samples (79%) were from tracheal aspirate (33%, *n* = 18), rectal swab, (22%, *n* = 12), and blood (24%, *n* = 13) cultures, while 11 (21%) came from wound (9%, *n* = 5), catheter tip (4%, *n* = 2), surgical drain (4%, *n* = 2), sputum (2%, *n* = 1), and urine (2%, *n* = 1) cultures (Figure 1B). Antibiotic resistance profiles of *S. marcescens* isolated from the abovementioned different sites showed that all strains were resistant to β-lactams antibiotics. In addition, colistin (CST) and tigecycline (TGC) non-susceptibility pattern of *S. marcescens* per site of isolation was statistically significant (*p* < 0.01) in several organs (tracheal aspirate, blood, rectal swab, and wound) when compared with amikacin (AMK), gentamicin (GEN), and ciprofloxacin (CIP) antibiotics (Figure 1C).

### Antimicrobial Resistance Profile and Genetic Markers for Antibiotic-Resistance and Virulence Patterns

**FIGURE 2:**
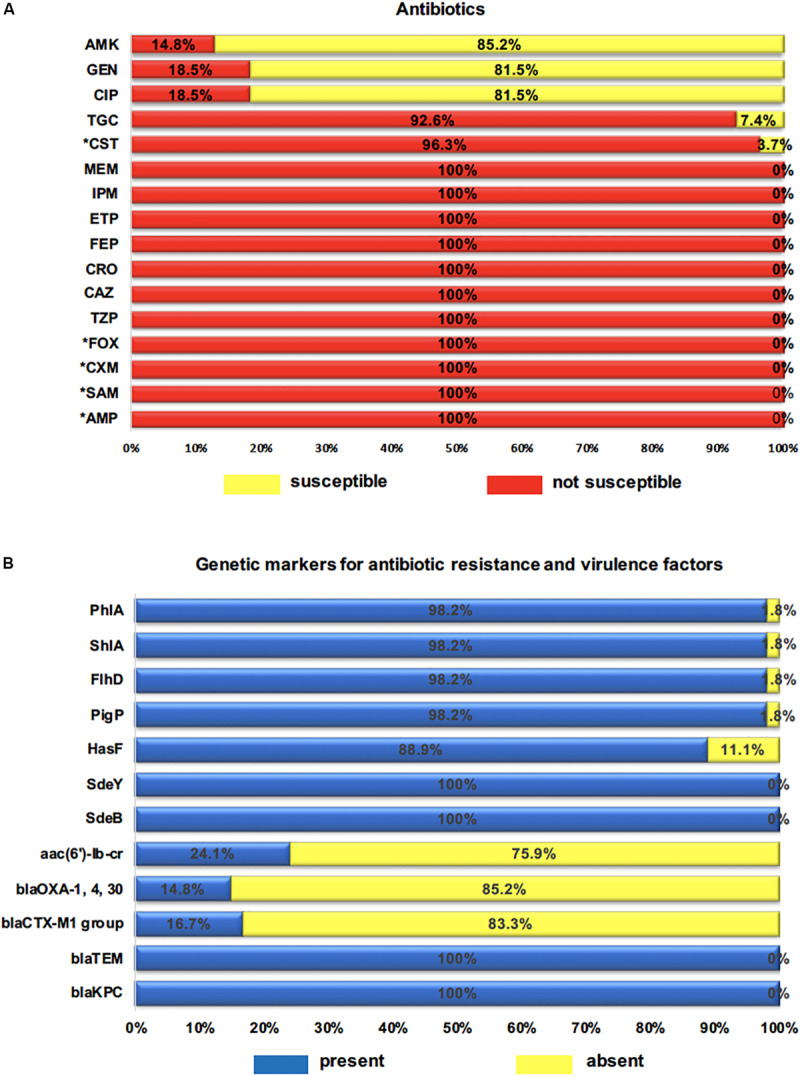
**(A)** Percentage of clinical isolates not susceptible to 16 antibiotics tested. AMP (ampicillin), SAM (ampicillin-sulbactam), TZP (piperacillin-tazobactam), CXM (cefuroximeaxetil), FOX (cefoxitin), CAZ (ceftazidime), CRO (ceftriaxone), FEP (cefepime), ETP (ertapenem), IPM (imipenem), MEM (meropenem), AMK (amikacin), GEN (gentamicin), CIP (ciprofloxacin), TGC (tigecycline), CST (colistin); * intrinsic resistance to antibiotics. **(B)** Percentage of genetic markers for resistance and virulence genes. *bla*_*KPC*_, *bla*_*TEM*_, *bla*_*CTX–M1*_, aac(6′)-Ib-cr: resistance genes; *Sde*B, *Sde*Y, *Has*F: efflux pump and outer membrane component genes; *Pig*P, *Flh*D, *Shl*A, *Phl*A: virulence genes.

*Serratia marcescens* strains showed high-levels of resistance to all β-lactams (100%, *n* = 54) (TZP, CAZ, CRO, FEP, ETP, IPM, MEM), including high-levels of intrinsic resistance to β-lactams (AMP, SAM, CXM, FOX) (100%, *n* = 54) and colistin (CST) (96.3%, *n* = 52). Resistance to tigecycline (TGC) *S. marcescens* was found in nearly all isolates (92.6%; *n* = 50). However, for the antibiotics classes fluoroquinolones (CIP) (81.5%, *n* = 44) and aminoglycosides such as gentamicin (GEN) (81.5%, *n* = 44), amikacin (AMK) (85.2%, *n* = 46) (Figure 2A), high susceptibility profile was detected. In contrast, MDR was observed in 24.1% (*n* = 13) of the isolates, and the most common MDR profile was related to β-lactams-glycylcycline-aminoglycosides-quinolone (14.8%, *n* = 8), followed by β-lactams-glicylcycline-quinolone (5.6%, *n* = 3), and (β-lactams-glicylcycline-aminoglycosides 3.7%, *n* = 2).

All 54 tested isolates harbored KPC-carbapenemase (*bla*_*KPC*_) and ESBL (*bla*_*TEM*_) genes. The ESBL-encoding genes *bla*_*OXA–*__1_ was detected in 14.8% (8/54), and the *bla*_*CTX–M–*__1 group_ in 16.7% (9/54). However, the *bla*_*SHV*_ variants, *bla*_*IMP*_, *bla*_*OXA–*__48_, *bla*_*NDM*_, *bla*_*VIM*_, and *mcr*-1 genes were not detected. The *aac(*6′)-Ib-cr variant gene that can induce simultaneous resistance against aminoglycoside and fluoroquinolone was found in 13 (24.1%) strains. The RND pump efflux encoding genes *Sde*Y and *Sde*B were identified in all strains while the outer membrane component gene (*Has*F) was present in 48 (88.9%). Thus, the coexistence of *Sde*Y/*Has*F genes and *Sde*B/*Has*F was observed in 49 (88.9%) strains (Figure 2B). Finally, with the exception of one strain (*Sm*40), the virulence-associated genes *Pig*P, *Flh*D, *Shl*A, and *Phl*A were regularly distributed among *S. marcescens* strains, which were detected in 98.2% of all strains (Figure 2B).

### Resistance Phenotype-Genotype Correlation and Genetic Markers for Virulence Factors

**FIGURE 3:**
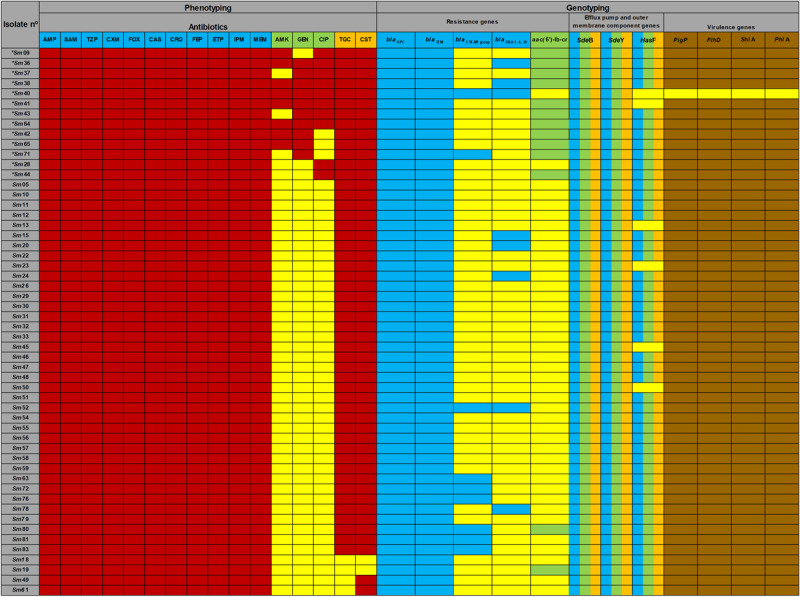
Phenotyping and genotyping of *Serratia marcescens* isolates. Sm represents *Serratia marcescens* and numbers represent identifications of strains. *Sm is classified as multidrug resistant (MDR) strains. AMP (ampicillin), SAM (ampicillin-sulbactam), TZP (piperacillin-tazobactam), CXM (cefuroximeaxetil), FOX (cefoxitin), CAZ (ceftazidime), CRO (ceftriaxone), FEP (cefepime), ETP (ertapenem), IPM (imipenem), MEM (meropenem), AMK (amikacin), GEN (gentamicin), CIP (ciprofloxacin). *Serratia marcescens* are intrinsically resistant to TGC (tigecycline) and CST (colistin). AMP, SAM, CXM, FOX, and CST antibiotics were not included in the MDR classification. The *bla*_*IMP*_, *bla*_*OXA–48*_, *bla*_*NDM*_, *bla*_*VIM*_, *bla*_*SHV*_ variants and mcr-1 genes were not detected. Blue box correlates with AMP, SAM, TZP, CXM, FOX, CAZ, CRO, FEP, ETP, IPM, MEM. Green box correlate with AMK, GEN, CIP. Multicolored box (*Has*F) correlates with CIP and TGC. Brown box shows number of *S. marcescens* caring virulence genes.

The correlation between the results of phenotypic and genotypic detection and the presence of virulence genes is shown in Figure 3.

All isolates carried *bla*_*KPC*_ and conferred resistance to all beta-lactam, including carbapenem antibiotics. Furthermore, all detectable bla genes in *bla*_*CTX–M–*__1_ *bla*_*OXA–*__1_, and *bla*_*TEM*_ group presented ESBL phenotype. Of the 13 isolates with *aac*(6’)-Ib-cr gene, 9 (69.2%) were non-susceptible to gentamicin, 7 (53.9%) to amikacin, and 8 (61.5%) to ciprofloxacin. Among the 49 (88.9%) *Has*F-positive isolates, 44 (81.5%) were non-susceptible to tigecycline.

### ERIC-PCR

**FIGURE 4:**
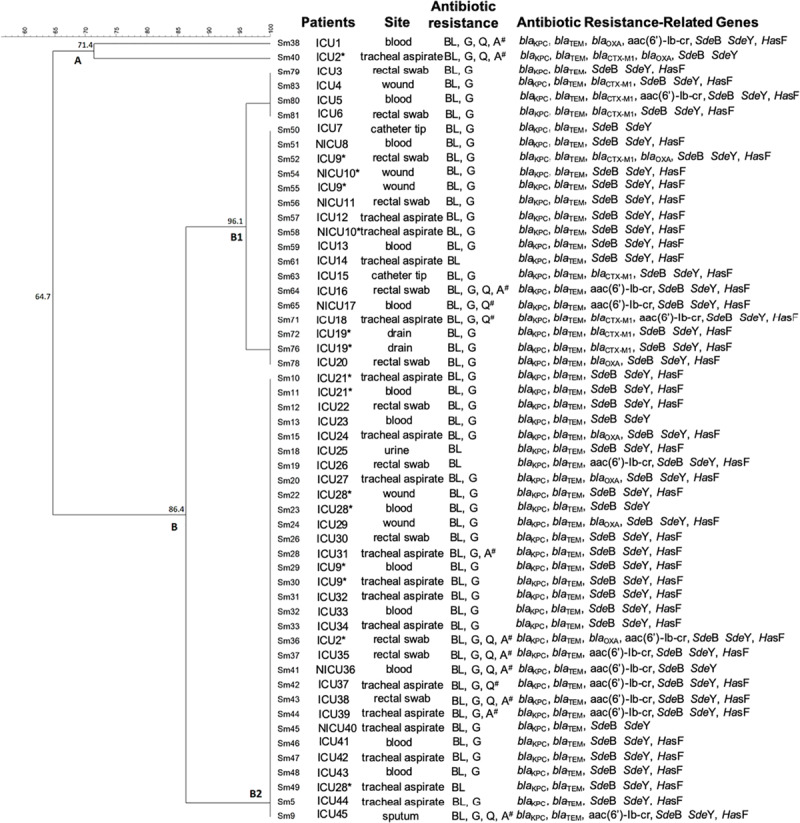
Dendrogram representing the genetic relationship between 54 strains of *Serratia marcescens* associated with patient localization, isolation site, antibiotic resistance, and virulence genes. ICU: Intensive Care Unit. NICU: Neonatal Intensive Care Unit. *Patients presenting *S. marcescens* in more than one infection site. ^#^ multidrug resistance (MDR) pattern.

The ERIC-PCR results indicated that the majority of the isolates presented a rate of genetic similarity above 85% (Figure 4). Almost all strains (96.3%) were grouped into a large cluster named B cluster, sharing 86.4% of genetic similarity. In addition, the B cluster was separated into two sub-clusters named B1, with 21 isolates, and B2, with 31 isolates, sharing a genetic similarity of 96.1% and 100%, respectively. Although the cluster B1 presented two subgroups with 4 and 17 isolates, they showed 100% genetic similarity in each one. Interestingly, two strains (*Sm*38 and *Sm*40) were grouped separately within the A cluster and presented 71.4% of genetic similarity (Figure 4).

## Discussion

*Serratia marcescens* is a prominent opportunistic pathogen that frequently causes infections in intensive care, surgical and dialysis units (Krishnan et al., 1991; Martineau et al., 2018). In Brazil, there are only few studies on *S. marcescens* (Ribeiro et al., 2013; Silva et al., 2015). Therefore, we here describe the presence of MDR *S. marcescens* isolates producing KPC-carbapenemase (*bla*_*KPC*_) and extended spectrum beta-lactamase (*bla*_*TEM*_, *bla*_*CTX–M–*__1 group_ e *bla*_*OXA–*__1_, _4 and 30_) in the state of Tocantins, Brazil. Tocantins, located southeast of the Northern Region, is the newest state of Brazil and shares borders with six states presenting intensive migration flow.

*Serratia marcescens* were isolated mainly from male patients with 60 or more years of age, similarly to previous studies that demonstrated advanced age male patients as presenting a higher risk of contracting *S. marcescens* infections (Ulu-Kilic et al., 2013; Kim et al., 2015; O’Horo et al., 2017). Samples with higher amounts of *S. marcescens* were those from tracheal aspirate, followed by blood, rectal swab, and wounds. Our findings corroborate studies by Kim et al. (2015) and Liou et al. (2014) that reported the respiratory tract as the main route of infection for *S. marcescens*. Other studies have also reported *S. marcescens* in other sites as bloodstream (Seeyave et al., 2006) and wounds (Us et al., 2017), demonstrating the versatility of these strains in colonizing the host and affecting a wide variety of physiological system.

In addition to the intrinsic resistance to the antibiotics AMP, SAM, CXM, FOX, and CST, we found multidrug-resistant (MDR) *S. marcescens* isolates to beta-lactam, glycylcycline, and/or aminoglycoside and quinolone group antibiotics. This is in line with other studies that have also reported MDR *S. marcescens* mainly to beta-lactam, aminoglycoside, and quinolone antibiotics groups (Stock et al., 2003), in hospital environment (Merkier et al., 2013), and particularly in critically ill patients and neonatal intensive care units (Maragakis et al., 2008). We also observed a significant resistance to colistin and tigecycline in several colonization sites, as shown by a previous study (Silva et al., 2017).

All the *S. marcescens* strains tested here were resistant to β-lactams, including carbapenems antibiotics. Resistance to carbapenems used to be uncommon among *Serratia* species (Stock et al., 2003), but many resistant strains have now emerged throughout the world (Lin et al., 2016). Although few studies have related resistance to carbapenems in *S. marcescens* in Brazil (Milisavljevic et al., 2004; da Costa Guimarães et al., 2013; Ribeiro et al., 2013), Silva et al. (2015) obtained similar results. They have isolated *S. marcescens* resistant to imipenem, meropenem and ertapenem in samples from different infection sites of ICU patients in another Brazilian locality. Both results are troubling. Infections caused by carbapenem-resistant bacteria often do not respond to conventional treatment (Okoche et al., 2015), as produced carbapenemases hydrolyze not only carbapenems but also penicillins, cephalosporins and monobactams (Queenan and Bush, 2007). The most common carbapenem resistance of *S. marcescens* in Brazil is due to the production of carbapenamases, especially the KPC-2 type (da Costa Guimarães et al., 2013; Ribeiro et al., 2013), that is encoded by the gene *bla*_*KPC–*__2_.

*Enterobacteriaceae*, such as *S. marcescens*, have the genes *bla*_*TEM–*__1_ and *bla*_*SHV–*__1_; these genes express classical class A beta-lactamases, encoded by plasmid that hydrolyze first generation penicillins and cephaloporins (Bush, 2010). We found gene *bla*_*TEM*_ in all isolates while gene *bla*_*SHVvariants*_ was not detected. It is noteworthy that even though *S. marcescens* also carries the gene *bla*_*CTX–M*_ (Yu et al., 2003; Kim et al., 2005; Tenover et al., 2013), few of our strains had the gene. Some strains also carried the genes *bla*_*OXA–*__1_, _4_ and _30_, that have been reported in few studies in Brazil or in other countries, either alone or associated with extended spectrum beta-lactamases genes (ESBL) (*bla*_*TEM*_, *bla*_*SHV*_ e *bla*_*CTX–M*_) in *S. marcescens* strains. Although there are discrepancies in frequency rate and in genotyping of ESBL-producing *S. marcescens* (Cheng et al., 2006), the observed beta-lactam antibiotic resistance may have also been caused by the genes *bla*_*TEM*_, *bla*_*CTX–M*_, and *bla*_*OXA*_, since the production of broad-spectrum beta-lactamases enzymes (TEM-1, TEM-2, SHV-1, OXA-1) generate resistant to ampicillin, ticarcillin, piperacillin, piperacillin/tazobactam and cephalosporin antibiotics, and the enzymes CTX-M have hydrolytic activity against cefotaxime (Levy and Marshall, 2004; Sugumar et al., 2014).

In our study, most strains of *S. marcescens* were only sensitive to aminoglycosides (gentamicin and amikacin) and fluoroquinolone (ciprofloxacin). Aminoglycosides are the oldest antibiotics that have been used less frequently in the last years, thus possibly preserving activity against some resistant bacteria that cause difficult to cure infections (Falagas et al., 2008; Gad et al., 2011). The observed low resistance to ciprofloxacin (18.18%) is in agreement with the results obtained by Sheng et al. (2002) who observed 20–30% resistance to quinolone in *S. marcescens* isolates. However, it is important to consider that *S. marcescens* is highly adaptable, so rates of resistance to fluoroquinolones diverge considerably among institutions (Young et al., 1980; Mahlen, 2011; Sader et al., 2014), including within Brazilian ones.

Resistance to fluoroquinolones may be caused by alterations in the target enzymes DNA gyrase and topoisomerase IV, and by acquisition of the transferable plasmid-mediated quinolone resistance (PMQR) determinants qnr, qepA, *aac*(6’)-Ib-cr, and oqxAB (Veldman et al., 2011; Poirel et al., 2012; Moradigaravand et al., 2016). The gene *aac*(6′)-Ib-cr, a variant gene of the aminoglycoside acetyltransferase, was also present in most of strains that presented fluoroquinolone and/or aminoglycoside resistance. This finding is consistent with others studies that have shown that *aac*(6′)-Ib-cr may be associated with antibacterial resistance against fluoroquinolone and aminoglycoside (Kim et al., 2009, 2011) antibiotics.

Three RND-type efflux have been reported in *S. marcescens*, namely *Sde*AB (Kumar and Worobec, 2005a), *Sde*CDE (Kumar and Worobec, 2005a; Begic and Worobec, 2008), and *Sde*XY (Chen et al., 2003). *Sde*AB and *Sde*XY interact with *Has*F (an outer membrane component, *Tol*C homolog gene) contributing to resistance against a wide variety of antimicrobial agents (Begic and Worobec, 2008; Hornsey et al., 2010). Although we did not analyze the genes *Sde*A e *Sde*X, the genes *Sde*B and *Sde*X were present in all strains and the gene *Has*F was also found in most strains. Drug extrusion by efflux pumps as *Sde*AB-*Has*F comprises one of the main mechanisms for fluoroquinolones antibiotic resistance (Dalvi and Worobec, 2012). Additionally, Hornsey et al. (2010) has shown the intrinsic activity of the *Sde*XY-*Ha*sF efflux pump is responsible for the lower susceptibility to ciprofloxacin. Thus, in addition to the presence of the genes *aac*(6′)-Ib-cr [associated with plasmid-mediated quinolone resistance (PMQR)], the genes *Sde*B, *Sde*Y, and *Has*F that encode RND-type efflux pump may have contributed to the observed ciprofloxacin resistance.

Our data shows high prevalence of tigecycline-resistant *S. marcescens* strains and *Sde*Y and *Has*F genes. The reduced sensitivity of *S. marcescens* to tigecycline may be related to the up-regulation of the *Sde*XY-*Has*F efflux pump (Hornsey et al., 2010). Although the gene *Sde*X was not analyzed, our findings strongly suggest that these genes may be responsible for the high tigecycline resistance.

Many bacteria produce virulence factors as hydrolytic enzymes and toxins that enable host invasion, bacterial proliferation and inhibit host defense mechanisms, sometimes resulting in host death (Aggarwal et al., 2017). *S. marcescens* strains were also evaluated for the presence of toxin genes *Shl*A and *phl*A, and all but the *Sm*40 strain carried these genes. Our findings are in agreement with other studies that reported the presence these genes in *S. marcescens* (strain SEN) (Aggarwal et al., 2017). *Flh*DC has been proposed as a regulator controlling flagellum biogenesis, biofilm formation, cell septation and expression of virulence factors during swarming (Givskov et al., 1995; Fraser and Hughes, 1999; Chilcott and Hughes, 2000; Lin et al., 2010). In our study, the presence of *Flh*C was not analyzed but the gene *Flh*D was present in almost all isolates. *S. marcescens* produces biosurfactant serrawettin and red pigment prodigiosin used in surfaces colonization (Hejazi and Falkiner, 1997). *Pig*P is a positive regulator of prodigiosin production that regulates swarming and hemolysis through serratamolide production (Fineran et al., 2005; Shanks et al., 2013). In our study, *Pig*P gene was found in almost all isolates (98.15%). Overall, our results suggest that the combination of these virulence genes could have contributed to the pathogenicity of *S. marcescens* strains.

The dendrogram based on ERIC-PCR fingerprint analysis demonstrated that the vast majority of isolates are closely related, sharing a genetic similarity of 86.4% (except for two strains). In addition, many isolates showed 100% similarity to each other. A study conducted by Ferreira-Firmo et al. (2019) evaluated nine *S. marcescens* from different clinical sources and three hospitals in Northeast Brazil showed a greater genetic diversity among the studied strains. Lin et al. (2016) studied 83 carbapenem-resistant *S. marcescens* isolates recovered from Zhejiang Provincial 501 People’s Hospital, China, from which they found 63 blaKPC-2 positive strains sharing nine different profiles. Our results demonstrate the predominance of few genetic profiles grouped together, with similarity above 85%, indicating that, although bacteria have been isolated from different patients and devices, the circulating *S. marcescens* in this hospital is highly genetically related.

Our study has some limitations worth noting. There was a high number of *S. marcescens* isolated from rectal swabs and tracheal aspirate, and both cultures are recommended for surveillance in ICU and NICU patients. However, microbiological reports sent to LACEN were not clear regarding how many of these samples were analyzed for both clinical and surveillance purposes. This study was further limited by the duration of the research, which was relatively short (2014–2015), and by conventional phenotypic and genotypic techniques, that have their own particular strengths and limitations in detecting MDR strains. Nonetheless, we intend to extend the analysis period of *S. marcescens* isolates, and whole genome sequencing to type relevant MDR strains must be performed. We expect that this study broadens our understanding on epidemiology, antibiotic resistance, and putative virulence factors of these strains, and provides relevant information for the prevention and management of *S. marcescens* infections.

In conclusion, *S. marcescens* represents a problem for public health (Kurz et al., 2003) and the resistance pattern exhibited by clinical isolates along with the transmission to other clones show the importance of researching factors associated with the increase in frequency and/or emergence of infections caused by *S. marcescens* in Brazilian hospitals. The occurrence of antibiotic-resistant bacteria considerably varies according to country, region and susceptible population, and the mitigation of this problem in ICUs is especially associated with actions to control the spreading of such drug-resistant bacteria (Ko et al., 2002; Hou et al., 2015). Thus, it is crucial to eliminate sources of resistance development and associated reservoirs as well as to overtake standardized sanitation and enforce mandatory notification to gather important data for continuous risk assessment evaluation and effective decision making to control these species in hospital environments.

## Data Availability Statement

The datasets generated for this study can be found in the GenBank database. Genes and their respective accession numbers: blaCTX – MK576103; blaKPC – MK576104; blaOXA – MK576105; blaTEM – MK576106; SdeB – MN583232; SdeY – MN583233; HasF – MN583234; aac(6′)-Ib-cr – MN583235; FlhD – MN583236; PigP – MN583237; ShlA – MN583238; PhlA – MN583239). Access to genetic heritage was approved by the National System for the Management of Genetic Heritage (SisGen n° AFF27ED).

## Ethics Statement

The studies involving human participants were reviewed and approved by the Committee of Ethics in Human Research of the Federal University of São Carlos (no. 1.088.936). In this work, all *Serratia marcescens* and the anonymous archival data related patient age, gender, and sample type were obtained from the Central Laboratory of Public Health of Tocantins (LACEN/TO, data’s owner). Permission to conduct the present study was obtained from the Health Department of the State of Tocantins (Secretaria da Saúde do Estado do Tocantins – SESAU) and LACEN/TO. Patient consent was not required, since the data presented in this study do not relate to any specific person or persons. Written informed consent from the participants’ legal guardian/next of kin was not required to participate in this study in accordance with the national legislation and the institutional requirements.

## Author Contributions

RF, GR, MD, MO-S, MB, and M-CP performed the experiments. FG and EL aided with statistical analysis. EC aided with the sequencing analysis and the sequence submission to the NCBI platform. M-CP conceived and supervised the study. M-CP, AC, IM, and AP-S wrote the manuscript and analyzed the data. MO-S performed ERIC-PCR.

## Conflict of Interest

The authors declare that the research was conducted in the absence of any commercial or financial relationships that could be construed as a potential conflict of interest.

## Footnotes

1 https://blast.ncbi.nlm.nih.gov/
