# Supplementary material for: Characterization of KPC-Producing Serratia marcescens in an Intensive Care Unit of a Brazilian Tertiary Hospital
Source: Front Microbiol. 2020 May 20;11:956. doi: 10.3389/fmicb.2020.00956 (PMC7326048; doi:10.3389/fmicb.2020.00956)
Supplement: Supplementary file 1 [file Data_Sheet_1.PDF]

Figure S1. PCR amplification of resistance gene in *Serratia marcescens*. 1% Ethidium bromide-stained agarose gel. M-1kb DNA ladder. The number in each lane of the electrophoresis gel image identifies the patient's number.

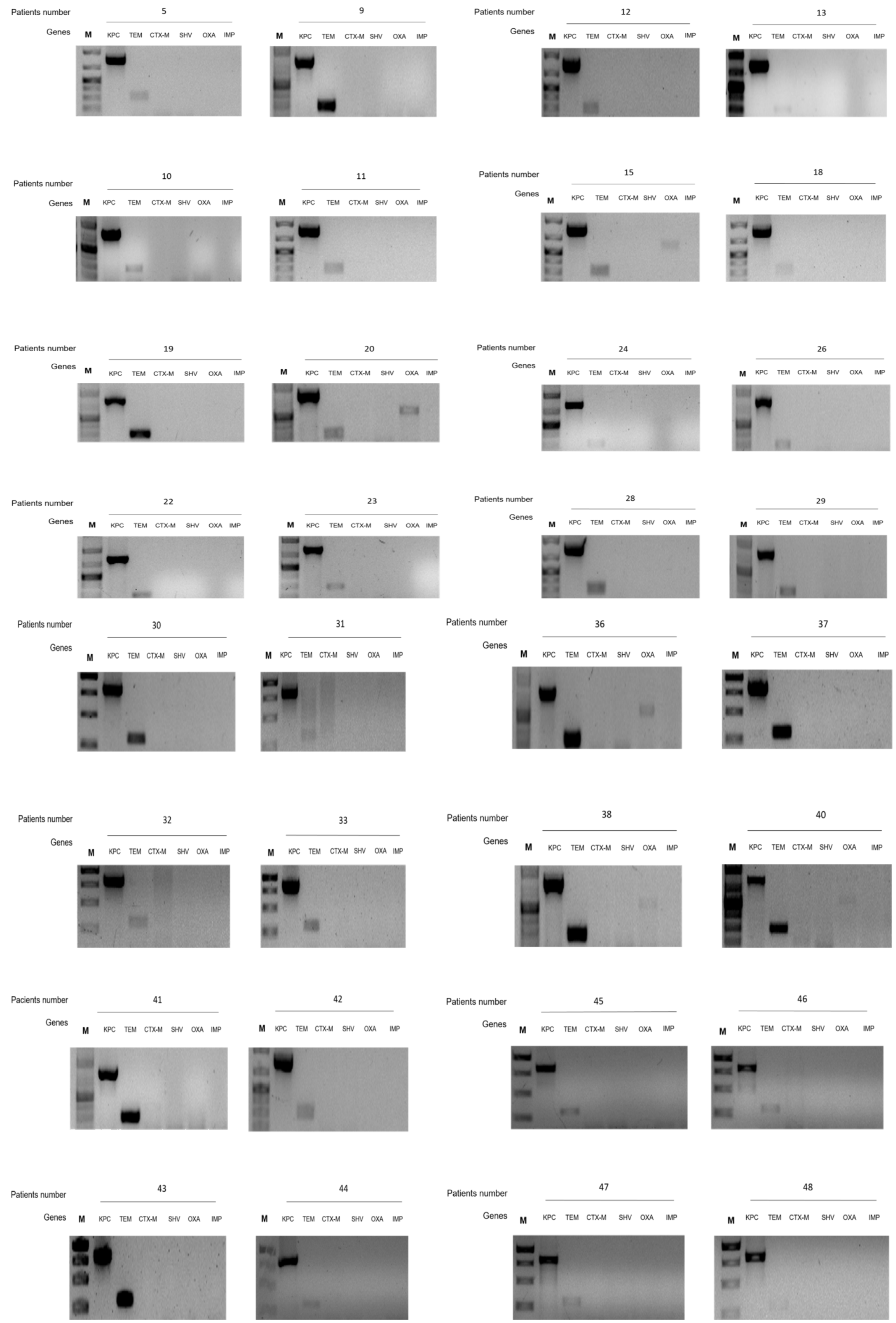

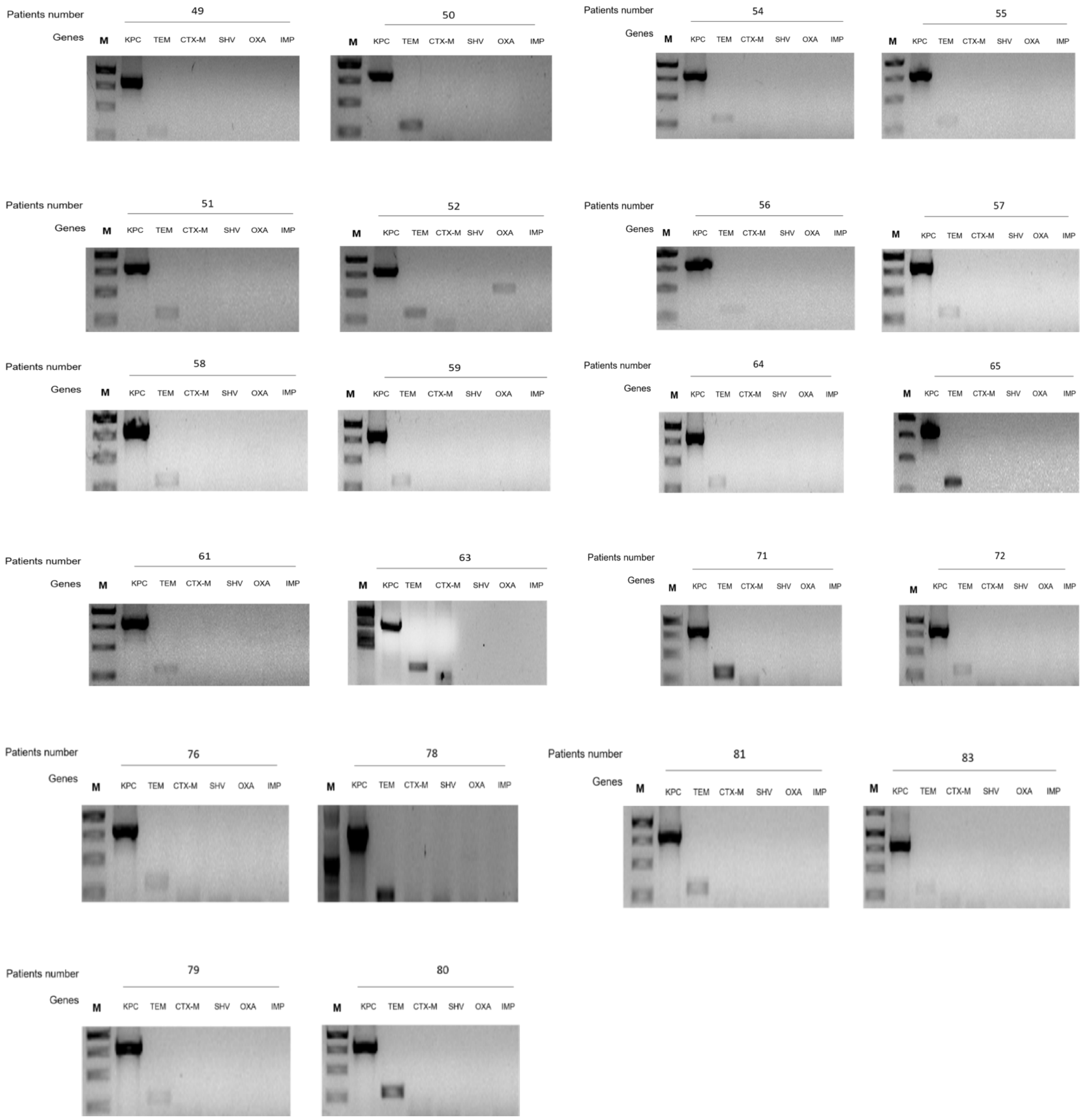

Patients number
Genes
49
M KPC TEM CTX-M SHV OXA IMP
50
M KPC TEM CTX-M SHV OXA IMP
Patients number
Genes
54
M KPC TEM CTX-M SHV OXA IMP
55
M KPC TEM CTX-M SHV OXA IMP
Patients number
Genes
51
M KPC TEM CTX-M SHV OXA IMP
52
M KPC TEM CTX-M SHV OXA IMP
Patients number
Genes
56
M KPC TEM CTX-M SHV OXA IMP
57
M KPC TEM CTX-M SHV OXA IMP
Patients number
Genes
58
M KPC TEM CTX-M SHV OXA IMP
59
M KPC TEM CTX-M SHV OXA IMP
Patients number
Genes
64
M KPC TEM CTX-M SHV OXA IMP
65
M KPC TEM CTX-M SHV OXA IMP
Patients number
Genes
61
M KPC TEM CTX-M SHV OXA IMP
63
M KPC TEM CTX-M SHV OXA IMP
Patients number
Genes
71
M KPC TEM CTX-M SHV OXA IMP
72
M KPC TEM CTX-M SHV OXA IMP
Patients number
Genes
76
M KPC TEM CTX-M SHV OXA IMP
78
M KPC TEM CTX-M SHV OXA IMP
Patients number
Genes
81
M KPC TEM CTX-M SHV OXA IMP
83
M KPC TEM CTX-M SHV OXA IMP
Patients number
Genes
79
M KPC TEM CTX-M SHV OXA IMP
80
M KPC TEM CTX-M SHV OXA IMP
