# Supplementary material for: Characterization of KPC-Producing Serratia marcescens in an Intensive Care Unit of a Brazilian Tertiary Hospital
Source: Front Microbiol. 2020 May 20;11:956. doi: 10.3389/fmicb.2020.00956 (PMC7326048; doi:10.3389/fmicb.2020.00956)

Figure S2. PCR amplification of antibiotic-resistance and virulence genes *in Serratia marcescens*. 1% Ethidium bromide-stained agarose gel. M-1kb DNA ladder. The number in each lane of the electrophoresis gel image identifies the patient's number.

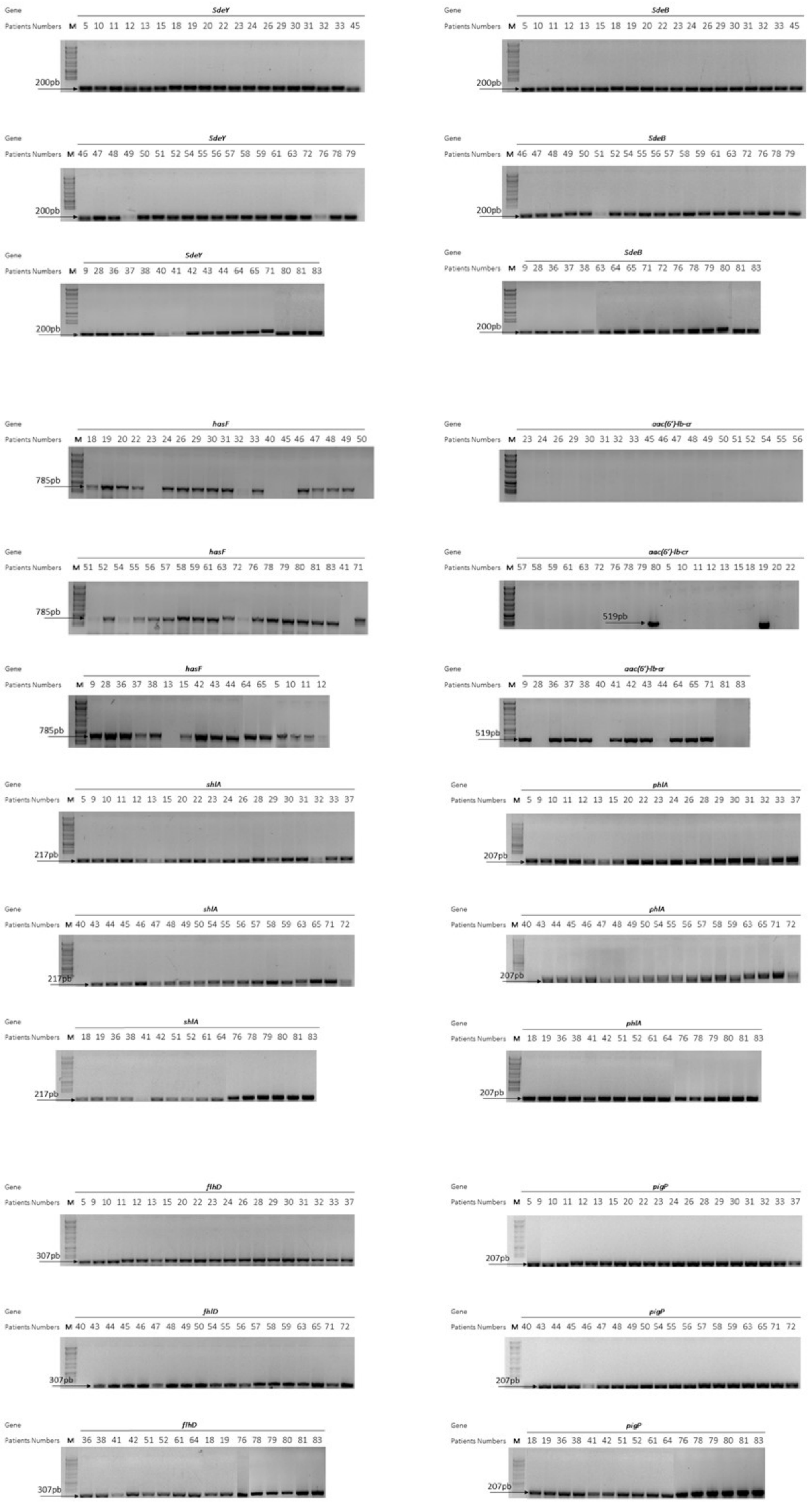

Supplement: Supplementary file 2 [file Data_Sheet_2.PDF]
